# Proteomics of Bronchoalveolar Lavage Fluid Reveals a Lung Oxidative Stress Response in Murine Herpesvirus-68 Infection

**DOI:** 10.3390/v10120670

**Published:** 2018-11-27

**Authors:** Eric Bortz, Ting-Ting Wu, Parthive Patel, Julian P. Whitelegge, Ren Sun

**Affiliations:** 1 Department of Biological Sciences, University of Alaska Anchorage, Anchorage, AK 99508, USA; 2 Department of Molecular & Medical Pharmacology, David Geffen School of Medicine, University of California, Los Angeles, CA 90095, USA; twu@mednet.ucla.edu; 3 Center for Molecular Biology and German Cancer Research Center (DKFZ), University of Heidelberg (ZMBH), 69120 Heidelberg, Germany; p.patel@dkfz-heidelberg.de; 4 The Pasarow Mass Spectrometry Laboratory & the Jane and Terry Semel Institute for Neuroscience and Human Behavior, David Geffen School of Medicine, University of California, Los Angeles, CA 90095, USA; jpw@chem.ucla.edu

**Keywords:** murine herpesvirus-68, MHV-68, bronchoalveolar lavage fluid, BAL, proteomics, oxidative stress

## Abstract

Murine herpesvirus-68 (MHV-68) productively infects mouse lungs, exhibiting a complex pathology characteristic of both acute viral infections and chronic respiratory diseases. We sought to discover proteins differentially expressed in bronchoalveolar lavage (BAL) from mice infected with MHV-68. Mice were infected intranasally with MHV-68. After nine days, as the lytic phase of infection resolved, differential BAL proteins were identified by two-dimensional (2D) electrophoresis and mass spectrometry. Of 23 unique proteins, acute phase proteins, vitamin A transport, and oxidative stress response factors Pdx6 and EC-SOD (Sod3) were enriched. Correspondingly, iNOS2 was induced in lung tissue by seven days post-infection. Oxidative stress was partly a direct result of MHV-68 infection, as reactive oxygen species (ROS) were induced in cultured murine NIH3T3 fibroblasts and human lung A549 cells infected with MHV-68. Finally, mice infected with a recombinant MHV-68 co-expressing inflammatory cytokine murine interleukin 6 (IL6) showed exacerbated oxidative stress and soluble type I collagen characteristic of tissue recovery. Thus, oxidative stress appears to be a salient feature of MHV-68 pathogenesis, in part caused by lytic replication of the virus and IL6. Proteins and small molecules in lung oxidative stress networks therefore may provide new therapeutic targets to ameliorate respiratory virus infections.

## 1. Introduction

Respiratory virus infections have the potential to cause significant lung pathology including acute respiratory distress syndrome (ARDS). In addition to the continual burden of disease from respiratory viruses such as influenza types A and B, respiratory syncytial virus (RSV), parainfluenza viruses, adenovirus, recently emerged coronaviruses responsible for Middle East (MERS-CoV) and severe acute (SARS-CoV) respiratory syndromes, H5N1 and H7N9 pathogenic avian influenza viruses, pandemic swine-origin (H1N1) influenza, and human metapneumovirus target the human lungs [1,2,3,4,5,6,7]. Co-morbid, underlying pulmonary medical conditions including asthma, chronic obstructive pulmonary disease (COPD), and tuberculosis (TB) are associated with severe respiratory virus infections [8,9,10,11]. Moreover, chronic pulmonary diseases such as asthma, COPD, and idiopathic pulmonary fibrosis (IPF) might have an infectious viral component to their onset and/or pathogenesis. The gammaherpesviruses Kaposi’s sarcoma-associated herpesvirus (KSHV/HHV-8) and Epstein–Barr virus (EBV) have been detected in the lung tissue of patients with IPF and acquired immune deficiency syndrome (AIDS)-related lymphocytic interstitial pneumonias [12,13,14]. In contrast, a murine gammaherpesvirus is protective in mice challenged with lethal influenza A virus, suggesting immunomodulatory roles for gammaherpesviruses in the pathogenesis of lung infections [14]. Thus, the application of new genomics technologies for understanding respiratory virus infections under isogenic host conditions might yield deeper insight into the pathological changes that occur in virus infection of the mammalian lung, and provide targets for diagnostic biomarkers or therapeutic intervention.

Murine gammaherpesvirus-68 (MHV-68) infects the lungs of wild and laboratory mice, productively infecting type I and II alveolar epithelial cells, macrophages, and dendritic cells (DCs) [15,16]. MHV-68 exhibits characteristics of both acute and chronic respiratory pathogens. Even though MHV-68 can inhibit type I interferon secretion by DCs [17], the lungs of mice infected by the virus eventually exhibit discernable pathology, including usual interstitial pneumonia (UIP) with diffuse alveolar damage (DAD), and infiltration of inflammatory cells [16,18,19]. MHV-68 infection induces pro-inflammatory chemokines including MCP-1, MIP-1a, MIP-1b, IP-10 and RANTES [20] in the lung, and interleukin-6 (IL6) and type II interferon (IFN-gamma in the draining (mediastinal) lymph nodes) [21]. Acute infection is resolved and lytic replication of the virus is cleared from the lungs in a CD8+ cytotoxic T-lymphocyte (CTL-) and type I alpha/beta-interferon receptor (IFNAR)-dependent manner [22,23]. Clearance also requires CD80/CD86 antigen presentation [24], IFN-γ [25], and some degree of CD4+ T-cell help reliant on PKCθ [26]. However, as gammaherpesvirus infection progresses into latency, MHV-68 DNA remains detectable in the lung parenchyma, recapitulating a chronic disease characterized by increased interstitial collagen deposition, immune deregulation, and fibroblast proliferative events similar to those thought to occur early in the development of idiopathic pulmonary fibrosis (IPF). This chronic pathology phenotype is more clearly observed under experimental conditions where the lungs have been insulted prior to infection by an agent that induces pulmonary fibrosis [27], such as fluorescein isothyocyanate (FITC), or in mice deficient in immune responses, such as interferon gamma receptor-null mice [15].

The pro-inflammatory cytokine IL6 is thought to be involved in the host response to respiratory infections. IL6 is upregulated in ARDS caused by bacterial pneumonias in human patients [28], and in LPS-treated human lung cells infected with RSV [29]. While no differences in viral life cycle, cytokine profiles, nor cytotoxic T-cell responses were observed in MHV-68 infection of IL6-deficient mice, IL6 does appear to regulate natural killer (NK) cells responding to MHV-68 infection [30]. Interestingly, KSHV also encodes a viral homologue of IL6 (vIL6) with distinct signaling activity during lytic infection [31,32]. To study the role of exogenous expression of this cytokine in gammaherpesvirus infection of the lung, we constructed a recombinant MHV-68 virus expressing murine IL6.

Proteomics analysis of bronchoalveolar lavage (BAL) fluid has emerged as a new approach to understand pathophysiological events occurring in acute, infectious, malignant or chronic obstructive lung diseases, both in human patients and animal models [33,34]. BAL is a rich source of information about lung cytokine and chemokine responses, inflammatory proteins, and immune cells present or infiltrating into the alveolar lumen. In proteomics studies, proteins isolated from BAL fluid have been typically analyzed by isoelectric focusing followed by two-dimensional electrophoresis (IEF/2DE), or by multidimensional protein identification technology (MudPIT), allowing for the identification of differentially-expressed proteins secreted or released via pathological processes into the alveolar lumen. These studies have begun to add a new dimension into the characterization of human or mouse physiological responses to acute lung injury (ALI) [33], acute respiratory distress syndrome (ARDS) [35,36], and diffuse interstitial lung diseases including IPF [37], COPD [38], and oxidative or toxicological damage to the lung [39,40]. In animal models of infectious diseases, BAL has revealed lung cytokine and chemokine profiles and immune cell populations in response to virus infections [15,20,41]. For a monkeypox virus infection model in macaques [42] and three pathogens in mouse infection models, RSV [43], *Staphylococcus aureus* [44,45], and *Klebsiella pneumoniae* [46], proteomics analyses of BAL have identified inflammatory proteins and revealed commonalities in infectious pulmonary pathophysiology. Analysis of mouse BAL by IEF/2DE showed a suppression of antioxidant and oxidative stress proteins during RSV infection [43]. However, no analysis using differential IEF/2DE proteomics in MHV-68 infection of the mouse lung have been published to date.

As many human viruses infect the lung, understanding the proteins present in BAL using MHV-68 as a model may uncover novel aspects of the mammalian host’s response to pulmonary viral infections. Using proteomics, we have identified mouse BAL proteins that are differentially up-regulated by virus infection and overexpression of a immunomodulatory cytokine (IL6). Proteins involved in the acute phase response, oxidative stress responses, and vitamin A signaling were salient in the MHV-68 infected lung. Interestingly, these proteins are induced by nine days post-infection (d.p.i.), as the initial phase of MHV-68 infection resolves and lytic replicating virus is cleared from the lungs by T-cell mediated host responses [20,23]. The experimental protocol herein demonstrates the feasibility of differential BAL proteomics to characterize less abundant, highly regulated host factors in BAL fluid.

## 2. Materials and Methods

### 2.1. Viruses and cell Cultures

Wild-type (WT) MHV-68, MHV68/IL6, and red fluorescent protein (RFP)/MHV-68 viruses in this study were all titered by plaque overlay assay on BHK21 cells as previously described [47,48]. Recombinant viruses were generated by co-transfection of MHV-68 genomic DNA and a PCR-generated cDNA encoding the gene to be inserted flanked by MHV-68 sequences corresponding to the MHV-68 genome. MHV68/IL6 virus was generated by homologous insertion of murine cDNA encoding interleukin-6 (IL6) driven by a cytomegalovirus (CMV) immediate early (IE) promoter–enhancer into an intergenic locus near the 5′ end of the MHV-68 genome [49]. The RFP/MHV-68 virus was generated in a similar manner whereby a cDNA encoding RFP driven by CMV IE promoter–enhancer was inserted into the ORF28 locus. The ORF28 gene is dispensable for infection of cultured cells and *Mus musculus* models of MHV-68 infection [50]. Recombinant viruses were selected by plaque-purification, viral DNA was purified and screened for cDNA insertion into expected loci by PCR and restriction fragment digestion followed by Southern blotting, as has been described [48,49,51]. During lytic infection in NIH3T3 cells, expression of IL6 from the MHV68/IL6 virus was confirmed by Western blotting and ELISA; for RFP/MHV-68, expression of RFP was observed by epifluorescent microscopy. To probe for reactive oxygen species (ROS), murine NIH3T3 or human A549 cells were infected with RFP/MHV-68 at a multiplicity of infection (m.o.i) of 1 or 5 and at 4 h or 20 h post-infection (h.p.i.), cells were rinsed in cold 1 × phosphate-buffered saline (PBS), incubated for 5 min at 37 °C in the dark in 1 × PBS containing 5 µM 5/6-carboxy-2′,7′-difluorodihydrofluorescein diacetate (H_2_DF_2_DA), a compound that exhibits superior photostability compared to other fluorescein derivatives (Invitrogen, Carslbad, CA, USA), washed in 1 × PBS, and then imaged in an epifluorescent microscope. ROS-inducing compounds H_2_O_2_ or paraquat (10 µM) were employed as positive controls for H_2_DF_2_DA fluorescence. For examining ROS effects on viral titer, NIH3T3 cells were infected with RFP/MHV-68 (m.o.i. = 0.25) in the absence or presence of 1 mM soluble glutathione (GSH) or 2–25 µM paraquat in media. After 20 h, culture supernatants were diluted 1/2, 1/10, or 1/100, used to re-infect fresh NIH3T3 cells, and RFP fluorescence observed 20 h.p.i. by epifluroescence microscopy using a Zeiss Axiovert epifluorescence microscope (C. Zeiss AG, Oberkochen, Germany).

### 2.2. Mouse Infections with MHV-68 and MHV68/IL6 Viruses

All in vivo mouse experiments were conducted at the University of California, Los Angeles (UCLA) in a dedicated animal facility under approved protocols, following ethical guidelines for laboratory animals in research by the Animal Research Committee at the University of California, Los Angeles (IACUC protocol number # 1999-058; Approved 1 Jan. 1999; renewed 2004–2006). Twelve-week old male C57/BJ6 mice (Charles River Laboratories, Wilmington, MA, USA) were anesthetized with 0.1 mL (100 mg/kg) ketamine by intraperitoneal (i.p.) injection, and then inoculated with 20 µL DMEM (6 mice) or infected intranasally (i.n.) with 5 × 10^5^ pfu of WT MHV-68 (6 mice) or MHV68/IL6 (6 mice) virus diluted in 20 µL DMEM. Mice in each experimental group were housed separately until sacrifice at 6 or 9 d.p.i., when at each timepoint, 3 mice in each experimental group were anesthetized and sacrificed under anesthesia by i.p. injection of 0.1 mL ketamine. Mice were subsequently dissected for bronchoalveolar lavage (BAL) thrice with 1.4 mL sterile 1 × PBS via a rounded 21G syringe inserted by tracheotomy and affixed with suturing thread. Separately, 2 more mice in the DMEM and in each infected group were sacrificed at 7 d.p.i. for whole-lung harvest with snap-freezing of tissue in liquid N_2_, and determination of viral titer and gene expression as described [47,49]. BAL fluid was centrifuged immediately (2000× *g*, 15 min., 4 °C) to separate soluble, supernatant phases and the cell/debris pellet. Supernatants were kept at −80 °C until processing. Cell pellets were resuspended in 50 µL 0.5% FBS DMEM containing 1 mM EDTA, and monocytes in 5 µL aliquots were counted by trypan blue exclusion test with a hemocytometer. Aliquots of cell fractions (5 µL) were also analyzed by thin smear on poly-lysine coated glass slides followed by fixation and eosin/hematoxylin staining with Hema3 (Thermo Fisher Scientific, Waltham, MA, USA) according to the manufacturer’s instructions, and light microscopy (Olympus Corp., Tokyo, Japan).

### 2.3. BAL Fluid Processing

Aliquots of BAL fluid supernatants were used for Sircol collagen assay, viral DNA detection by quantitative PCR, and protein detection by Western blotting. For each experimental infection (MHV-68 and MHV68/IL6), one of the three BAL samples containing significant numbers of erythrocytes was deselected. To have sufficient protein for resolution by proteomics methods, the remaining 2 BAL fluid supernatants were pooled for each experimental condition, precipitated in 95% acetone at −20 °C for 2 h, centrifuged (4 °C, 15 min, 20,000× *g*), and then resuspended in binding buffer. To reduce abundant immunoglobulins and albumin, Aurum column binding and elution (Bio-Rad, Hercules, CA, USA) was done according to the manufacturer’s instructions. Eluents were re-concentrated and desalted by 4:1 acetone (95%, ice-cold) precipitation for 2 h, centrifuged (4 °C, 15 min, 20,000× *g*), and resuspended in isoelectric focusing (IEF) buffer. A Bradford assay was used to quantify protein concentration prior to and post-processing [52], and SDS-PAGE with SYPRO-Ruby staining was used to observe depletion of abundant albumin bands.

### 2.4. Sircol Collagen Assay

For lung tissue collagen assay, 0.05 g lung tissue was homogenized in 0.5 M acetic acid (1 mL) containing 7.5 mg pepsin, and rotated for 24 h at 4 °C. Samples were briefly centrifuged to pellet debris, and 100 µL of each supernatant was assayed for collagen by Sircol assay as described by the manufacturer (BioColor Ltd., Carrickfergus Belfast, UK). For measuring soluble collagen in BAL fluid, aliquots of 25 µL were subjected to Sircol assay. Collagen concentration was determined by absorbance at 540 nm in a spectrophotometer and titration according to standard curves generated for lung tissue and BAL fluid.

### 2.5. Catalase Assay and Immunoblotting

NIH3T3 cells were lysed in passive lysis buffer and protein content was normalized by a Bradford assay as described previously [52], and lysates were subjected to a catalase activity assay (Sigma, St. Louis, MO, USA) according to the manufacturer’s instructions. A standard curve was generated with controlled quantities of H_2_O_2_. H_2_O_2_ treatment for 24 h yielded only a minimal induction of catalase activity in this assay. For Western blots, protein lysates separated by SDS-PAGE were Western blotted and probed with specific polyclonal anti-ORF65/M9 anti-sera or anti-catalase antibody (Calbiochem, San Diego, CA, USA) with HRP-linked secondary and electrochemiluminescent detection as described [48].

### 2.6. PCR

For quantitative RT-PCR, total RNA was isolated from mouse lungs 7 d.p.i. and reverse transcribed into cDNA as described [47,49]. Primers to specific murine genes (described in Supporting Information Table S2) were used to amplify transcript cDNA and relative transcript copies determined by the ΔΔC_T_ method with an actin internal control by SyberGreen (Applied Biosystems, Carlsbad, CA, USA) real-time detection on a LightCycler thermocycler (Roche, Indianapolis, IN, USA). Significance of relative gene expression was determined by an unpaired, 2-tailed *t*-test. Viral DNA representing viral genome copy number was determined for each mouse BAL sample by qPCR with primers specific to MHV-68 genomic ORF65/M9 or ORF57 loci as previously described [48,51].

### 2.7. IEF, 2D-PAGE, Spot Mapping and Densitometry

Eluted BAL proteins (300 µL) were resuspended in IEF buffer containing ampholytes covering the pH 3–10 range (Bio-Rad, Hercules, CA, USA). Samples passively loaded on rehydrated, immobilized 11 cm nonlinear pH 3–10 gradient IPG strips (Bio-Rad, Hercules, CA, USA) and then focused by *p*I for 18 h ramping over 6 h to a maximum current of 70,000 V-h in a Protean IEF Cell (Bio-Rad, Inc., Hercules, CA, USA). Strips were re-equilibrated for 30′ in DTT and then iodoacetamide buffers and proteins separated by mass in a denaturing 8–16% gradient Criterion 2D-PAGE (Bio-Rad, Inc., Hercules, CA, USA). Two-dimensional gels were briefly incubated in 10% methanol/5% acetic acid, rinsed in ddH_2_O, and stained for 3 h with SYPRO-Ruby (Invitrogen, Carlsbad, CA, USA). Gels were imaged under UV light and analyzed to identify differentially-expressed protein spots. Proteins resolved in the pH 4–7 range were sufficiently separated for spot mapping across gels using an integrated ProteomeWorks *PD Quest 7.1* imager and software (Bio-Rad, Carlsbad, CA, USA) with manual spot validation. Spots were quantified by peak cross-sectional densitometry using *ImageQuant* (GE Healthcare, Piscataway, NJ, USA), and normalized to an average of oxytocin-receptor (spot 13) and a common major form of eluted albumin (spot 5) relative to gel image background density. A total of 89 abundant differential spots across the three experimental conditions were excised and in-gel digested in Trypsin Gold MS (Promega, Madison, WI, USA), and alkylated peptides were extracted, dried and stored at −80 °C as described previously [53] for mass spectrometry identification.

### 2.8. Mass Spectrometry

Tryptic peptide digests of proteins were separated on a reverse phase column and identified by tandem micro-LC/MS-MS and in some cases, by MALDI-TOF mass spectrometry, with sample handling as described previously [54,55]. BSA (5 pmol) digested in Trypsin Gold was used to generate positive control spectra for LC/MS-MS and MALDI-TOF experiments, respectively. Briefly, MS-MS spectra were captured on an AB Sciex Qstar quadrapole XL hybrid TOF LC/MS-MS (Applied Biosystems, Foster City, CA, USA) with tandem peptide ion fragmentation running in Information Dependent Acquisition (IDA) mode. Peptide and fragment a-, b- and y-series ions spectra were analyzed by *Mascot* software (Matrix Sciences, Boston, MA, USA) with peptide tolerance set at <0.5 Da, MS/MS tolerance < 0.8 Da, charge states + 1/ + 2/ + 3/ + 4, 1 tryptic digest miss allowed, oxidation of Cys and Met, with peptide identification by search against the predicted mouse proteome at NCBI and EBI reference databases. From tryptic digests of excised spots, 44 yielded peptide data identifying 23 unique proteins. Positive identification cutoffs were determined on a case-by-case basis with expectation scores < 10^−2^ (*p* < 0.05, for 20 hits), or *p* < 0.1, for 3 hits, considering multiple peptide hits and supporting MALDI-TOF data in assignment. Another 7 spots did not meet a significance cutoff or poorly matched predicted *p*I and MW, including annexin A5, hemoglobin fragment, triose phosphate isomerase, matrix metalloproteinase 8, serpin b 3d, and collagens I and VI *(p* > 0.10).

For MALDI-TOF, aliquots of peptide digests were mixed with 200× proportion of α-cyano FHSA matrix dissolved in 70% acetonitrile and 0.1% TFA and spotted with laser ionization and data capture with a low mass gate (500 Da) on an AB Sciex Voyager MALDI-TOF running *PD Quest* software (Applied Biosystems, Foster City, CA, USA). MALDI peptide data were searched against the mouse proteome using *Aldente* software [56], with predicted *p*I and molecular mass data estimated from 2D-PAGE spots.

### 2.9. Bioinformatics Analyses

Functional enrichment among the set of proteins discovered in enriched BAL fluid was analyzed by *Ingenuity Pathways Analysis* (IPA 7.6, Ingenuity Systems Corp., Redwood, CA, USA) as described [57]; IPA categories were tested for significance by a Benjamini–Hochberg test for false discovery [58]. BAL protein functions were also analyzed by Database for Annotation, Visualization, and Integrated Discovery (DAVID) algorithms [59] to assess significant Gene Ontology (GO), InterPro (IPR), and Protein Information Resource (SP_PIR) annotations. A subnetwork of oxidative stress-associated molecules was discovered and extracted using IPA with manual literature curation to construct a network model [60]. Amino acid sequences of human and mouse TNFAIP8 family proteins were obtained from the UniProt database and CLUSTAL multiple sequence alignments performed and formatted using MAFFT FFT-NS-2 v5.731 [61].

## 3. Results

### 3.1. Recovery and Characterization of BAL Fluid from Mouse Lungs Infected with MHV-68

**Figure 1:**
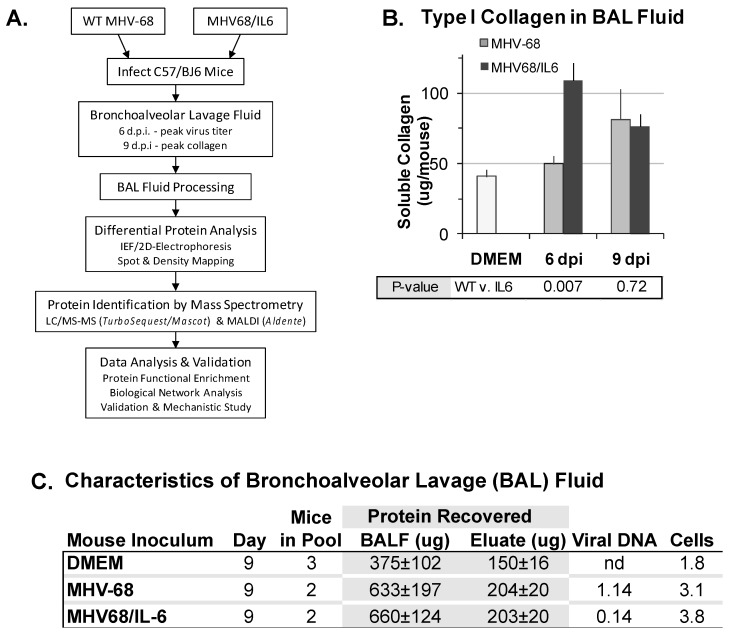
Analysis of bronchoalveolar lavage (BAL) fluid from murine herpesvirus-68 (MHV-68) infection of the mouse lung. (**A**) Overview of differential proteomics analysis of proteins induced in BAL by virus infection. Wild-type (WT) MHV-68 or MHV68/IL6 virus infection, BAL fluid processing, protein spot identification, and protein data analysis scheme. BAL fluid processing removed cells, immunoglobulins, excess albumin, and salts, enriching the recovered eluent for less-abundant proteins. (**B**) Collagen production in MHV-68 infection of the mouse lung is exacerbated by lytic expression of interleukin-6 (IL6). C57/BJ6 mice were mock infected with DMEM or infected intranasally with 5 × 10^5^ pfu of WT MHV-68 or MHV68/IL6 virus. Type I collagen in BAL fluid in MHV-68 and MHV68/IL6 infection was measured at indicated times post-infection by Sircol assay; *p*-value of fold induction estimated from unpaired, 2-tailed *t*-test; error bars represent standard error on the mean (S.E.M.) (**C**) Characteristics of bronchoalveolar lavage (BAL) fluid. BAL fluid recovered 9 days post-infection (d.p.i.) from C57/BJ6 mice inoculated as above was processed to remove cells, salts, abundant serum proteins and immunoglobulins. Average protein concentration was measured by Bradford assay (±1 s.d.); eluate exhibited significant differences (*p* < 0.05, unpaired, 2-tailed *t*-test) between MHV-68 and DMEM and MHV68/IL6 and DMEM. Average viral DNA copy number (1 × 10^5^ cp) in BAL in 3 mice for each condition measured by qPCR, and average mononuclear cellularity (1 × 10^5^ cells) measured by trypan blue hemocytometry; differences not significant. IEF = isoelectric focusing.

An unexpected observation regarding MHV-68 infection of laboratory mice was that MHV-68 infection could exacerbate pulmonary fibrosis [15,27,62,63,64,65]. Thus, we also sought to identify proteins induced by MHV-68 infection accompanied by co-expression of murine IL6, a pro-fibrotic cytokine. C57/BJ6 mice were inoculated intranasally (i.n.) with DMEM, or infected with a high titer of WT MHV-68 or a recombinant MHV-68 virus co-expressing murine interleukin-6 (IL6) from a constitutive promoter. To discover secreted or extracellular proteins in virus infection of the mouse lungs, we developed an experimental procedure to analyze the BAL fluid proteome (Figure 1A). At six d.p.i. and nine d.p.i., BAL fluid was collected and analyzed for cells, protein, soluble collagen, and viral DNA content. At six d.p.i., MHV68/IL6 showed significantly more soluble type I collagen in BAL fluid than WT MHV-68 infection; by nine d.p.i., soluble type I collagen was significantly higher in BAL fluid from both WT and MHV68/IL6 infection in comparison to the uninfected control (Figure 1B). Subsequent analysis focused on the nine d.p.i. timepoint, for which soluble type I collagen levels in BAL were similar between the WT virus and MHV68/IL6, and good resolution of proteins by IEF/2DE was achieved. Both protein concentration and mononuclear cellularity in BAL fluid were substantially higher in infected vs. uninfected mice at nine d.p.i., and viral DNA was detected (Figure 1C). MHV-68 viral capsid antigen ORF65/M9 was also present in clarified BAL fluid (Figure S1B). Differences in extracellular virion DNA or DNA from damaged cells in the lung (Figure S1A), which are not a direct measurement of infectious virus titer, were not significant (*p* > 0.1, two-tailed *t*-test). Numbers of BAL mononucleocytes recovered (Figure 1C) was similar to a previous report of phenotypic characterization of mononuclear cell infiltrates (Figure S1C), chemokines and cytokines in MHV-68 infection of the lungs [20].

### 3.2. Differential Proteomics Analysis of BAL Fluid

**Figure 2:**
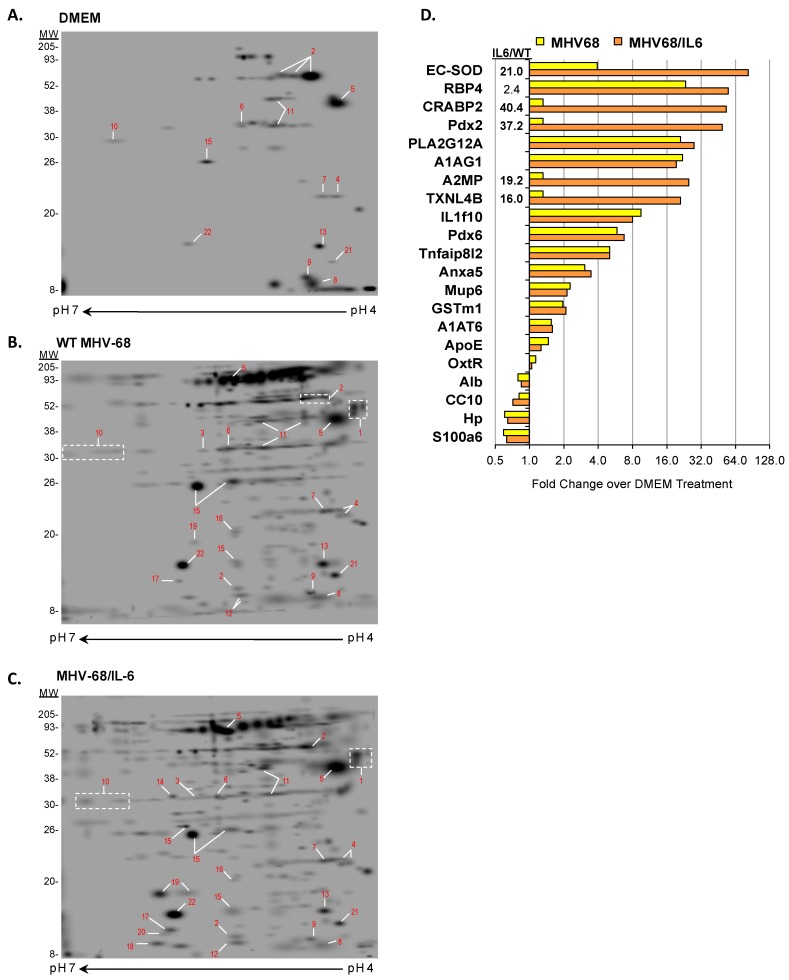
Enriched BAL proteome from mice infected with MHV-68 viruses. Mice were intranasally (i.n.) inoculated with DMEM (**A**), or infected i.n. with 5 × 10^5^ pfu of WT MHV-68 (**B**) or MHV68/IL6 virus (**C**). BAL was collected from mouse lungs 9 d.p.i., pooled, and processed to enrich for less abundant proteins as described in Methods. Eluted BAL proteins were separated by isoelectric focusing followed by 2D-PAGE and SYPRO-Ruby staining. Proteins resolved in the pH 4–7 range were sufficiently separated for spot identification (*red numbers*), excision, and tryptic digestion for protein identification by MALDI and/or LC/MS-MS. Spots were quantified by densitometry, normalized as described in Methods, and fold induction over orthologous spots in mock (DMEM) treatment is indicated (**D**). Significant fold induction (>2.0) of MHV68/IL6 over WT MHV-68 specified.

Recovered BAL fluid from nine d.p.i. was pooled for each experimental condition (three mice in DMEM, or two mice for WT MHV-68 and MHV68/IL6, respectively), after processing to remove cells and reduce abundant immunoglobulins, albumin, and salts. Reduction of the most abundant proteins in biofluids is a common approach for reducing proteome complexity to enrich less abundant but biologically interesting proteins [66]. Enriched BAL proteins were analyzed by comparative 2D gel electrophoresis (IEF/2DE) display (Figure 2). WT MHV-68 and MHV68/IL6 infection induced a considerably more complex proteome than DMEM-inoculated control mice. Prominent constitutive and differentially-expressed orthologous proteins were mapped and identified by LC/MS-MS and/or MALDI mass spectrometry. We mapped 39 spots in the pH 3–7 range, and identified 23 unique proteins, of which 20 proteins had high significance scores (*p* < 0.05), for example peroxiredoxin 6 (Pdx6, spot #15 in Figure 2; see Figure S2 for example of detailed peptide LC/MS-MS data), and three proteins (A2MP, OxtR, and Tnfaip8l2) had marginal scores (*p* < 0.1) for at least two peptide matches (Supporting Information Table S1). Even though viral ORF65/M9 antigen was detected by Western blot in clarified BAL fluid supernatants (Figure S1), peptides matching MHV-68 virion proteins [53] in enriched BAL fluid data did not reach the significance cutoff (*p* > 0.10). Of the proteins identified, 13 were induced by WT MHV-68 infection (six strongly), and five were markedly upregulated in the context of MHV68/IL6 (Figure 2D). Another four proteins showed a reduced abundance in the context of either virus infection in comparison to DMEM-treated or uninfected control mice (Figure 2D and Table S1).

### 3.3. Functions of Proteins Induced by MHV-68 in Lungs

While this survey is not a comprehensive list of BAL proteins [34,67], the proteins identified fell into four broad functional groups according to Gene Ontology (GO) classification and the scientific literature (Table 1): (i) acute phase response (APR) and inflammation, (ii) oxidative stress response, (iii) phospholipid metabolism and signaling, and (iv) molecular transport and serum proteins. Most of these proteins have been implicated in inflammation or lung diseases, and many have been identified in proteomics studies of BAL from human patients with ARDS [35,36,68], acute lung diseases [33], IPF [37], or proteomics analysis of serum from patients with severe acute respiratory syndrome (SARS) caused by SARS-coronavirus [69]. The MHV68/IL6 virus induced three antioxidant (thioredoxin-like 4B, peroxiredoxin 2, superoxide dismutase 3) and two acute phase (α2-macroglobulin, CRABP2) proteins substantially more than WT MHV-68. Accordingly, oxidative stress [70,71] and acute phase responses [72,73] have been shown to be regulated by IL6.

### 3.4. Functional Enrichment Analysis

**Figure 3:**
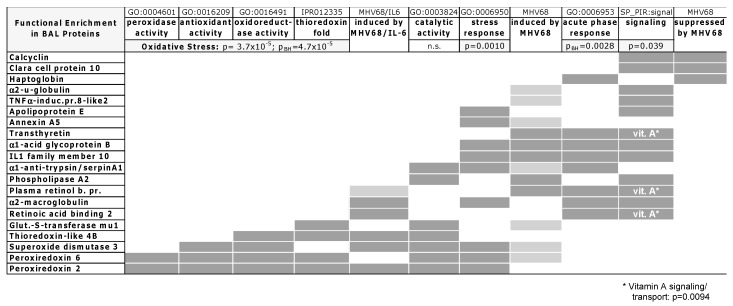
Functional enrichment matrix for identified BAL proteins. Protein functional enrichment for 20 out of 23 BAL proteins identified, clustered by Gene Ontology (GO), InterPro (IPR), and Protein Information Resource (SP_PIR) term annotation, Ingenuity Pathways Analysis (IPA), or regulation by WT MHV-68 (MHV68) and MHV68/IL6 (infex). Dark gray, inclusion in functional term or strong regulation. Light gray, weak induction by MHV-68 or MHV68/IL6. Proteins involved in vitamin A (vit. A *) signaling and transport were enriched within the signaling category with *p* = 0.0094. Significance estimated by *p*-values (*labeled* p) for enrichment in annotated functional categories; Benjamini–Hochberg (p_BH_) false discovery probability values for IPA category analysis. n.s. = not significant.

To gain a more systematic understanding of protein functions induced in response to MHV-68 infection of the lung, we undertook bioinformatics analyses to identify functional enrichment for the 20 of 23 significant or marginally significant proteins identified in MHV-68 BAL. Albumin, a reference serum protein, and protein fragments (Hydin and OxtR) were not included. Among BAL proteins, significantly enriched functional categories included physiological stress (*p* = 0.0010), oxidative stress annotations (*p* < 0.0001), and acute phase response (*p* = 0.0028) (Figure 3). Oxidative stress response proteins included reactive oxygen species (ROS) and detoxifying enzymes (peroxiredoxins, thioredoxins, and superoxide dismutase). Acute phase response (APR) proteins overlapped with other enriched functions, including physiological stress response and signaling (*p* = 0.039). Among signaling proteins, vitamin A (retinoic acid) binding was a significant function (*p* = 0.0094) for three proteins that were also induced in APR: CRABP2, transthyretin (TTR), and plasma retinol binding protein (RBP4). Proteins induced by WT MHV-68 infection were predominantly in the stress response, acute phase, signaling, and oxidative stress categories. Exogenous expression of IL6 in the context of MHV-68 infection primarily induced oxidative stress and APR proteins as well as vitamin A binding protein CRABP2; RBP4 was weakly induced in the context of IL6. Finally, signaling and APR proteins (calcyclin, Clara cell protein 10, and haptoglobin) were found to be less abundant in WT MHV-68 compared to the control (“suppressed by MHV68”, Figure 3) but clearly not oxidative stress proteins.

### 3.5. Acute Phase and Oxidative Stress Gene Expression in the MHV-68 Infected Lung

**Figure 4:**
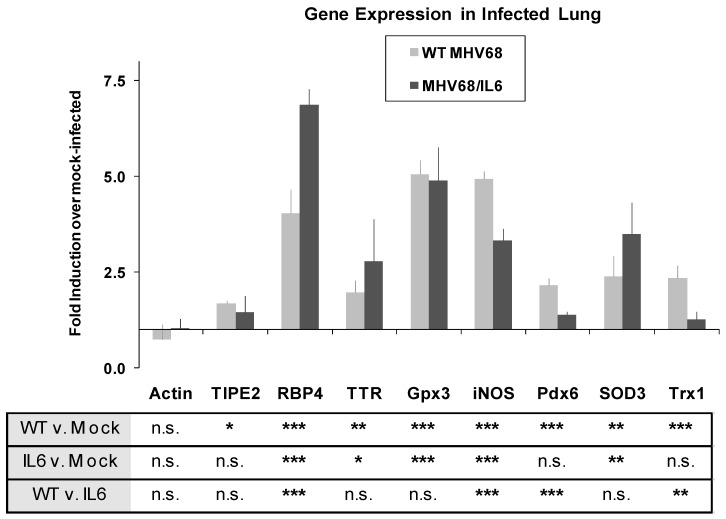
MHV-68 infection upregulates stress response genes in the lungs of infected mice. Mice were mock-infected, or infected i.n. with 2 × 10^5^ pfu of WT MHV-68 or MHV68/IL6. Total lung RNA was isolated 7 d.p.i., and qRT-PCR performed with specific primers to murine acute phase, immunomodulatory, oxidative stress response genes, and actin. *p*-values of fold induction estimated from unpaired, 2-tailed *t*-test (sub-table) with *p* < 0.01 (***), *p* < 0.05 (**), *p* < 0.1 (*), or *p* > 0.1 (n.s., not significant). Error bars represent S.E.M. *TIPE2*, Tnfaip8l2; *Gpx3*, glutathione peroxidase 3; *iNOS*, inducible nitric oxide synthetase; *SOD3*, (extracellular) superoxide dismutase 3; *Trx1,* thioredoxin 1; *RBP4, TTR, Pdx6*, as in Table S2.

To further investigate acute phase and oxidative stress responses in lung tissue, the expression of known host genes involved in these pathways was studied by quantitative real-time (qRT) PCR. Two mice for each condition were infected with WT MHV-68, MHV68/IL6, or mock (DMEM) inoculated, and RNA was extracted from total lung homogenates for qRT-PCR. By seven d.p.i., APR/vitamin A transport genes RBP4 and TTR were upregulated approximately four- and two-fold, respectively, with significantly higher induction by MHV68/IL6 for RBP4 (Figure 4). MHV-68 infection also generally induces oxidative stress genes in the lung by seven d.p.i. (Figure 4), including genes encoding lung antioxidant proteins Pdx6, EC-SOD, glutathione peroxidase 3 (Gpx3), and thioredoxin 1 (Trx1), as well as inducible nitric oxide synthetase (iNOS), a pro-inflammatory protein that is capable of generating reactive oxygen species (ROS) and reactive nitrogen species (RNS) as a byproduct of the production of NO messenger [74].

### 3.6. Lytic MHV-68 Infection Induces Oxidative Stress in Cultured Fibroblasts

**Figure 5:**
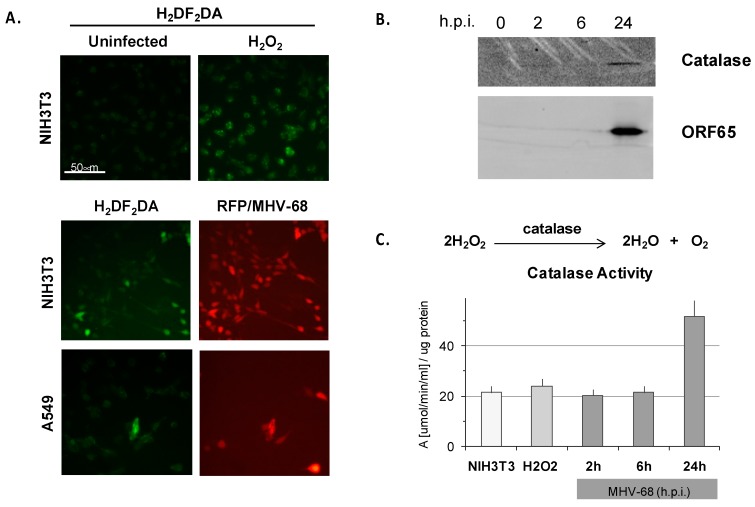
MHV-68 infection induces reactive oxygen species (ROS) in cultured cells. (**A**) Murine NIH3T3 fibroblasts or human lung A549 cells were infected (m.o.i. = 1) in triplicate with MHV-68 expressing red fluorescent protein (RFP/MHV-68, *red channel*). Supernatants were removed 20 h.p.i., and the cells were incubated with H_2_DF_2_DA and imaged by epifluorescence microscopy. Hydrogen peroxide (H_2_O_2_) was a control for induction of ROS leading to oxidative fluorescence of H_2_DF_2_DA *(green channel)*. (**B**) NIH3T3 cells infected in triplicate with WT MHV-68 were lysed at the indicated times, proteins separated by SDS-PAGE, and Western blots probed for catalase protein and MHV-68 lytic antigen (ORF65) with specific antibodies and electrochemiluminescent secondary antibody detection. (**C**) Induction of ROS measured by catalase activity assay. NIH3T3 cells in triplicate were untreated, treated with H_2_O_2_ for 20 h, or infected with WT MHV-68 for the times indicated; cells were lysed and aliquots measured for protein content by Bradford and catalase activity assays according to a standard curve; only for 24 h, *p* < 0.05, two-tailed *t*-test; error bars represent S.E.M.

To investigate the autonomous contribution of the MHV-68 lytic phase to oxidative stress in infected cells, we studied the role of the MHV-68 lytic phase in the production of ROS in murine NIH3T3 fibroblasts and human lung epithelioid A549 cells. As a control, we treated uninfected NIH3T3 cells with hydrogen peroxide, and induction of ROS was evident by oxidative green fluorescence of H_2_DF_2_DA (Figure 5A, upper panel). To examine whether ROS is induced by MHV-68 infection, sub-confluent NIH3T3 or A549 cells were infected with a recombinant MHV-68 virus expressing red fluorescent protein (RFP) from the ORF28 late locus, and then stained with H_2_DF_2_DA at 20 h.p.i. The majority of infected NIH3T3 or A549 cells (indicated by red fluorescence) exhibited a moderate to bright green H_2_DF_2_DA oxidative fluorescence, indicative of a high level of ROS (Figure 5A, lower two panels). Roughly 15% of infected NIH3T3 cells exhibited bright H_2_DF_2_DA oxidative fluorescence, indicative of a high level of ROS, by 20 h.p.i. (Figure 5A). ROS can lead to the generation of peroxides such as H_2_O_2_, which are reduced by the multi-subunit catalase enzyme. Indeed, catalase enzyme (Figure 5B) and catalase activity (Figure 5C) were upregulated in NIH3T3 cells infected with WT MHV-68 (m.o.i. = 1) by 24 h.p.i. In contrast, only basal catalase activity was observed early during MHV-68 infection at two h.p.i. and six h.p.i., analogous to simply adding excess H_2_O_2_ (Figure 5C). ROS did not accumulate by four h.p.i. even in a high titer infection (m.o.i. = 5), but did by 20 h.p.i. (Figure S3), suggesting that cytotoxicity associated with the late lytic cycle of virus infection is required for the generation of ROS. However, ROS induction itself seems to have little effect on MHV-68 infection; modulating cellular redox potential in infected NIH3T3 cells with sub-lethal doses of the oxidative stress inducer paraquat only weakly enhanced lytic expression of RFP/MHV-68, and quenching ROS with soluble glutathione had little discernable effect (Figure S3).

### 3.7. An Oxidative Stress Response Network Induced in Mouse Lungs by MHV-68 Infection

**Figure 6:**
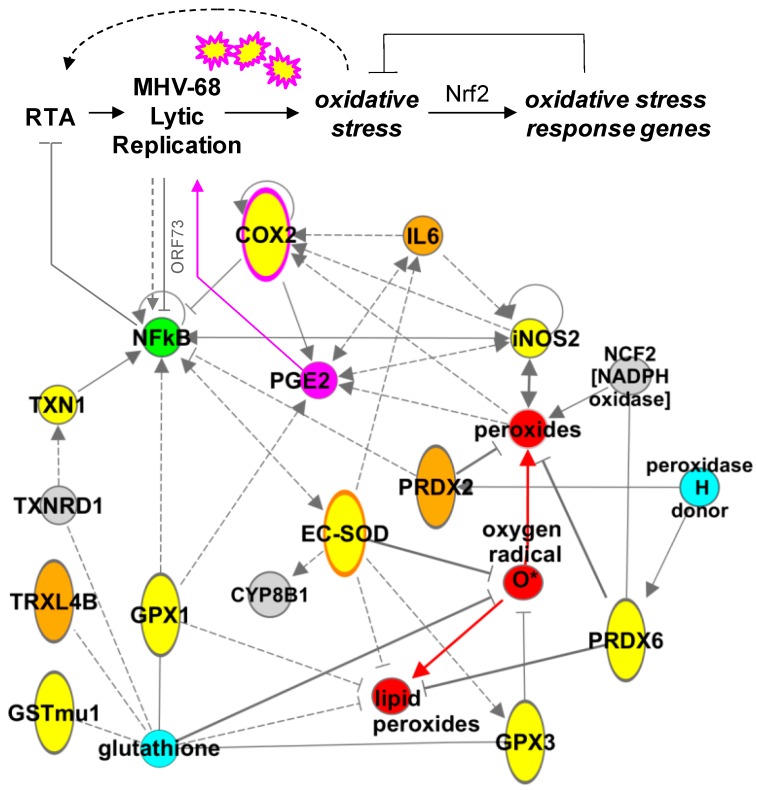
Oxidative stress network induced in mouse lungs by MHV-68. A sub-network of antioxidant proteins was extracted by IPA and manually curated. MHV-68 lytic infection induces reactive oxygen species (ROS) small molecules *(red)* that in turn induce oxidative stress response genes. Network model includes proteins induced in infected lungs by MHV-68 *(yellow)* or by IL6 in the context of MHV-68 infection *(orange* and *orange-border)*, proteins directly upregulated by MHV-68 lytic infection (*pink* and *pink-border*, [52]), and NF-kB, which has a complex interaction with MHV-68 lytic replication (*green*, [75]). Antioxidant co-factors *(aqua)*, other redox proteins *(gray)*, activating interactions *(arrows)*, indirect regulation *(dashed arrows and bars)*, and inhibitory interactions *(blocking bars)* indicate the potential complexity of even a small-scale redox network in vivo.

To gain a deeper understanding of the pathways induced in response to MHV-68 infection of the lung and co-expression of IL6, we used Ingenuity Pathways Analysis (IPA) to extract an oxidative stress and inflammatory response network centered on redox proteins identified in this study. While the number of proteins identified in our proteomics study (Table S1) was insufficient for de novo network discovery [60], analog curation using data from the Ingenuity KnowledgeBase and NCBI EntrezGene allowed synthesis of a model depicting regulatory interactions (i.e., activation, inhibition, etc.) among key molecules (Figure 6). A striking feature of the model is the multi-directional interaction between antioxidant proteins and transcriptional regulatory factors such as COX-2, NF-kB, and iNOS, all of which have been found to be important to gammaherpesvirus infections [25,52,75].

## 4. Discussion

We have undertaken a differential proteomics analysis of BAL fluid to gain insight into the pulmonary molecular pathology of respiratory virus infections in mice. As a tractable animal model of gammaherpesvirus infection, the pathogenesis and immune response to MHV-68 in the mouse lung has been a subject of considerable recent inquiry [16,18,19,20,21,23,47,64]. In such studies, BAL fluid has been used to analyze immune cell infiltration, cytokine/chemokine profiles, and chemotaxis activity, for example [15,20]. We found molecules in BAL fluid that provide additional insight as virus-induced lung injury is resolved, uncovering molecular details (i.e., host factors and pathways) of the host response to a virus in a quantifiable manner. Proteins induced by nine d.p.i. included oxidative stress response proteins, acute phase proteins, signaling molecules and transporters (Table 1). Functional category analyses indicated that redox, acute phase, and vitamin A proteins were significantly enriched in the subset of BAL proteins we identified (Figure 3), suggesting that these processes are induced by nine d.p.i. in MHV-68 infection of the mouse lung.

### 4.1. Effects of Co-Expressing IL6

As suggested by experiments in IL6-deficient mice [30], IL6 may play a role in inflammation and the development of lung pathology during MHV-68 infection rather than impacting viral replication. Instead of comparing BAL proteins between WT and IL6-deficient mice, we took a different approach to examine the effects of IL-6 by using an MHV68/IL6 virus that over-expresses this cytokine. Co-expression of IL-6 in MHV-68 infection of the mouse lung showed neither significant difference in replication kinetics nor whole lung viral titers in comparison to wild-type virus. However, MHV68/IL6 induced a subset of BAL proteins, including redox, acute phase, and vitamin A signaling/transport molecules (Figure 2D), as well as type I collagen (Figure 1B). IL6 gene expression is induced by NF-kB heterodimers, and IL6 in turn signals through an IL6 (CD126) receptor–gp130 co-receptor complex on a subset of B-cells to NF-IL6, a pro-inflammatory transcription factor [76,77]. In the lung, IL6 regulates natural killer (NK) cells responding to MHV-68 infection [30]. KSHV, a human gammaherpesvirus, encodes a viral IL6 homologue that can signal though the gp130 co-receptor found on a range of B-cells, independently of the cellular IL6 receptor [78]. The KSHV lytic transactivator replication and transcription activator (RTA) also activates the human IL6 promoter [79]. In addition, KSHV microRNAs (miRNAs) specifically induce IL6 and IL10 in macrophages [80]. Besides querying the effects of supranormal IL6 levels on infected lung pathophysiology, inclusion of the MHV68/IL6 virus in our BAL proteomics analysis allowed for the development of an analytical IEF/2DE method for differential protein discovery (Figure 2), demonstrating the potential utility of this approach for querying viral mutants.

### 4.2. Oxidative Stress Response Proteins Are Induced in MHV-68 Infection of the Mouse Lung

In the BAL proteome, host antioxidant and oxidative stress response proteins were upregulated by MHV-68 infection (Table 1), including Pdx6, EC-SOD, and a paralogue of GST (GSTm1). In whole lung tissue, genes encoding iNOS, extracellular glutathione peroxidase (Gpx3), and a thioredoxin (Trx1), were also induced (Figure 4). Co-expression of IL6 in MHV-68 infection further upregulated EC-SOD (Figure 2D), and induced another peroxiredoxin (Pdx2) and a thioredoxin paralogue (TXNL4B). Induction of antioxidant proteins suggests a pathophysiological response in the lungs to oxidative stress. Induction of oxidative stress has been found in experimental virus infections in vivo, including RSV in mice [43,81], and influenza virus infections in human epithelial cells, mice [82,83] and macaques [57]. Antioxidant proteins can protect lung tissue from oxidative damage, detoxify oxidized phospholipids, and reduce virus-associated ALI [83,84,85]. Oxidative stress induced in respiratory virus infections can also have pleiotropic effects on lung gene expression and inflammatory processes such as cytokine and chemokine production [57,82,86].

#### 4.2.1. Sources of Oxidative Stress

We found that MHV-68 infection of cultured NIH3T3 fibroblasts or lung-derived A549 cells induces ROS and catalase activity (Figure 5). Similarly, cultured cells infected with respiratory syncytial virus (RSV), rhesus monkey rhadinovirus (RRV, another gammaherpesvirus), or HSV-1 show increased oxidative stress [43,87,88,89]. Two genes upregulated by MHV-68, COX-2 [52] and iNOS (Figure 4), are capable of directly generating ROS as reaction byproducts [74,90]. Lytic MHV-68 infection proceeds under conditions of oxidative stress, as we found that treating infected cells with paraquat did not inhibit but rather mildly enhanced RFP/MHV-68 virus infection (Figure S3). In vivo, mice treated with NSAID-targeting COX-2 showed no differences in MHV-68 titers than controls ([52]). In contrast, cytotoxic T-lymphocyte (CTL) immune control of MHV-68 is impaired in mice deficient in iNOS, resulting in lethality [25]. While viral infection of type I and II lung epithelial cells likely contributes directly to the induction of oxidative stress, there may be other contributing factors in the alveolar microenvironment, such as degranulation of activated innate immune effector cells (alveolar macrophages, natural killer cells, and neutrophils). For example, the Ncf1/NADPH oxidase complex in neutrophils also significantly contributes to ROS and oxidation of phospholipids in lungs insulted with H5N1 highly pathogenic avian influenza (HPAI) [70].

#### 4.2.2. Oxidative Damage to Surfactant Phospholipids

Pulmonary surfactant lipids and proteins have roles in antiviral defense and inflammatory and immune responses against respiratory viruses such as influenza A viruses, RSV, and adenovirus [91]. Conversely, oxidative damage to phospholipids is implicated in ALI caused by viruses such as HPAI, SARS-CoV [70], and RSV [81]. Oxidized phospholipids that accumulate in HPAI and SARS-CoV infections also likely contribute to ALI and hypercytokinemia (“cytokine storm”) by signaling though toll-like receptor 4 (TLR4) and TRIF/TICAM1 in macrophages, activating NF-kB and inducing IL6 [70]. We found PLA2G12A, a secreted phospholipase A2 enzyme, highly upregulated in BAL from MHV-68 infected mice at nine d.p.i. (Table 1 and Figure 2D). Phospholipase A2 enzymes are involved in the degradation of damaged (oxidized) surfactant phospholipids including dipalmitoyl phosphatidylcholine (DPPC), a process often upregulated in lung injury. Phospholipase A2 is inhibited by abundant surfactant proteins including surfactant protein A (SP-A; [92]), and Clara cell protein 10 (CC10), which was downregulated in BAL from MHV-68 infection (Figure 2D). Another protein induced in BAL, Pdx6, may reduce oxidized phospholipids, including DPPC, that have been modified by ROS, allowing lipid recycling in type II epithelial cells or macrophages in the lungs [93,94,95]. Accordingly, surfactant protein expression is altered in chronic MHV-68 infection of interferon gamma receptor (IFNGR)-null mice that display a pathology reminiscent of IPF [15]. These finding suggest a role for lung surfactant lipids and lipid-associated proteins in the pathogenesis of MHV-68 in the lungs.

#### 4.2.3. Comparison to other Respiratory Diseases and Role of Nrf2

In contrast to MHV-68 infection, expression of antioxidant (oxidative stress response) proteins SOD1, GPx1, Pdx6, GSTmu1, and catalase were suppressed during RSV infection in the lungs of mice and human patients [43]. The antioxidant transcription factor Nrf2 was also suppressed in RSV infection, while Pdx2 was induced like in MHV-68 infection. The importance of the Nrf2-mediated response was illustrated in knockout mice, whereby Nrf2 protected lung cells from bronchopulmonary injury by RSV and influenza A virus [81,82]. Interestingly, a close association between oxidative stress and pro-fibrotic inflammation in the lung, marked by elevated Nrf2 expression, is well-established in human patients with IPF and/or interstitial pneumonia [96,97]. Human Pdx2 particularly has also been found upregulated in UIP/IPF lung tissue, in particularly in alveolar macrophages [98]. Thus, the role of Nrf2 in the induction of antioxidant defenses and pro-fibrotic pathophysiology of MHV-68 is in need of further investigation.

### 4.3. Modeling a Complex Relationship

A network of molecules intersects with these antioxidant proteins, including NF-kB, IL6, COX-2, iNOS, Nrf2, and small molecules, suggesting multiple points at which MHV-68 infection might generate an oxidative stress in the lungs (Figure 6). For example, while it is suspected that NFκB can be activated by oxidative stress in viral infections [81,82], the relationship between NF-kB signaling and gammaherpesvirus pathogenesis is complex and poorly understood. It has been reported that NFκB is activated by MHV-68 lytic replication [63], while paradoxically, NF-kB activation can also inhibit the initiation of MHV-68 lytic replication [75]. Regulation of NF-kB is an enriched function among cellular proteins interacting with MHV-68 proteins [99], and the lytic protein ORF73 promotes ubiquitination and degradation of p65/RelA [100]. Likewise, inhibition of NF-kB leads to upregulation of ROS and reactivation of latent KSHV by activating expression of the lytic transactivator protein, RTA [101]. Moreover, experimental inhibition of NF-kB blocks chemokine responses and development of pulmonary fibrosis in the lung in MHV-68 infection [63]. Functional genomics studies may provide additional molecular insight into virus–host interactions controlling MHV-68 infection and induction of oxidative stress [99].

#### 4.3.1. Acute Phase Response

Appearance of acute phase proteins in the serum is a hallmark of systemic inflammation resulting from infections, including bacterial sepsis, pneumonia, and human immunodeficiency virus (HIV-1) [102,103,104,105]. The release of acute phase proteins from liver hepatocytes or other tissues is dependent on IL6 and other cytokines [106,107]. The finding of acute phase proteins in BAL from MHV-68 infected mice indicate a systemic response to intranasal MHV-68 infection, or leakage of serum proteins into the pleural interstitial and alveolar lumen, consistent with previous findings of UIP pathology in MHV-68 infection [16]. We found acute phase-related proteins in BAL at nine d.p.i, including α1-antitrypsin (A1AT6), α2-macroglobulin (A2MP), α1-acid glycoprotein 1B (A1AG1/AGP), haptoglobin, and vitamin A transport molecules CRABP2, TTR, and RBP4 (Table 1 and Figure 3). AGP is immunomodulatory, induced in experimental pulmonary tuberculosis [108] and influenza [72] in mice. A1AT6 is a protease inhibitor induced by MHV-68, while the endopeptidase haptoglobin is suppressed (Figure 2D); along with type I collagen accumulation (Figure 1B), an anti-proteolytic lung tissue remodeling environment is apparent. IL6 has also been suggested to enhance the production of acute phase response proteins in virus infections [106]. Indeed, the protease inhibitor A2MP is induced in MHV68/IL6 infection (Figure 2D), consistent with higher type I collagen deposition. Finally, vitamin A (retinoic acid, RA) is a signaling molecule carried in the serum as all-trans retinol by an RBP4 and TTR dimer complex [109]. Vitamin A inhibits HSV-1 [110] and KSHV [111] replication in cell culture. Vitamin A can activate the immune response to infection in the respiratory tract, for example, by enhancing Th2 responses and IgA secretion in influenza virus infection of mice [112]. In the lungs, vitamin A counter-acts IL6 and protects against bleomycin-induced fibrotic lung injury [113,114]. The immunoregulatory function of vitamin A is not understood in MHV-68 infection.

#### 4.3.2. Other Immunomodulatory Proteins in BAL Fluid

One gene encoding an immune modulator, Tnfaip8l2, was induced in BAL by MHV-68 infection by nine d.p.i. (Table S2). While LC/MS-MS peptide data identification of this protein was of marginal significance (*p* < 0.1), the gene encoding Tnfaip8l2 was also weakly upregulated in lung tissue by seven d.p.i. (Figure 4). TNFα-interacting protein 8 members, including Tnfaip8l2 (TIPE2), form a conserved gene family in humans and mice (Figure S4) involved in immune homeostasis. TIPE2 downregulates inflammatory responses mediated by toll-like receptors (TLR), T-cell receptors (TCR), and NFκB signaling, which in turn promotes Fas-mediated apoptosis in lymphoid cells [115]. Except for Tnfaip8l2 and a weak match to annexin A5, we did not find other cell death regulators in BAL at nine d.p.i. Cell death in MHV-68 infection has been found to be mediated by CD8+ CTL in the lung [23], while a viral Bcl2 encoded in the MHV-68 genome blocks internal cell death mechanisms such as autophagy in infected cells [116].

### 4.4. Limitations of This Study

Our BAL processing protocol enriched for differentially-expressed proteins remaining after the reduction of abundant high-MW macromolecules, including albumin, immunoglobulins, and serum proteins (Figure 1C). While this approach allowed us to resolve less-abundant proteins, it likely missed potentially interesting proteins associated with the removed macromolecules. We also did not detect a high diversity of cytokines in post-processed BAL, possibly because of their relatively low abundances, interactions with antibodies or albumin, or failure to isolate highly basic proteins in the purification schema. Variant protocols enriching different BAL fractions, or using different BAL solvents, and new mass spectrometry technologies are in development.

## 5. Conclusions

### Experimental MHV-68 Infection of the Mouse as a Model for Lung Diseases

The induction of and responses to oxidative stress appear to be a common theme in the pathophysiology of interstitial lung diseases, including infections such as MHV-68 (this study and [15]), SARS-coronavirus [69], influenza A virus [70,82], RSV [43], and in chronic diseases such as COPD [117] and IPF [96]. Interestingly, a number of the gene products we identified as differentially regulated by MHV-68 infection in BAL were also found to be associated with these diseases (Table 1). Differential proteomics analysis of mouse BAL fluid opens a new window into understanding the pathogenesis of MHV-68 and other respiratory viruses, and MHV-68 models of chronic lung diseases such as IPF [15]. The proteins identified herein are potential biomarkers for pulmonary virus infections generating high levels of oxidative stress and aggravating other pathophysiological responses, such as acute phase (Figure 3) and surfactant lipid damage. We propose continuing application of differential BAL proteomics in conjunction with whole-lung genomics and proteomics analyses [57,118] to integrate a systems understanding of immune responses and virus-induced pathological changes to the pleura.

## Figures and Tables

**Table 1:** Functions of bronchoalveolar lavage proteins identified in MHV-68 infection. Functions of constitutive and differentially-expressed proteins identified by proteomics from BAL fluid from mock (DMEM), MHV-68 infected, and MHV-IL6 infected mice, with selected findings in lung pathophysiology, inflammation and infectious diseases

Spot ^a^	BAL Protein Identification	Symbol	GO:Terms ^b^	Function	Lung Disease Finding ^c^
**Acute Phase Response and Inflammation**					
1	α1-acid glycoprotein 1B	A1AG1	0002682	Lipocalin-like immune regulator	APR; TB; IAV
2	α1-anti-trypsin (serpin A1)	A1AT6	0004867	Serine-type endopeptidase inhibitor	APR; ARDS; COPD; SARS; IAV
3	α2-macroglobulin	A2MP	0004867	Serum-type endopeptidase inhibitor	APR; ARDS; IPF
11	Haptoglobin	Hp	0004252	Serine-type endopeptidase	APR; SARS
12	IL1 family member 10	IL1f10	0005152	IL1-receptor antagonist	APR; IPF
21	TNF α-induced protein 8-like 2	Tnfaip8l2	0050728	Negative immune regulator	Anti-proliferative
**Oxidative Stress Response**					
19	Superoxide dismutase 3 [Cu-Zn], ex.	EC-SOD	0006979	Response to oxidative stress	ARDS; NFκB; antioxidant
10	Glutathione-S-transferase, mu1	GSTm1	0004364	Response to oxidative stress	Antioxidant
14	Peroxiredoxin 2	Pdx2	0006979	Response to oxidative stress	ARDS; SARS; IAV; antioxidant
15	Peroxiredoxin 6	Pdx6	0000302	Response to reactive oxygen species	Sf-PhL; IAV; antioxidant
20	Thioredoxin-like 4B	TXNL4B	0030612	Thioredoxin activity	Antioxidant
**Phospholipid Metabolism and Signaling**					
8	Calcyclin	S100a6	0048146	Fibroblast proliferation	Growth factor
9	Clara cell protein 10	CC10	0019834	Phospholipase A2 inhibitor	Sf-PhL
13	Oxytocin receptor	OxtR	0004990	Oxytocin receptor activity	
16	Phospholipase A2, secreted	PLA2G12A	0004623	Phospholipase A2 activity	SARS; IAV; Sf-PhL
12	Hydrocephalus-inducing protein	Hydin	0003341	Movement of tracheal cilia	
**Molecular Transport/Serum**					
5	Albumin	Alb	0006810	Molecular transport in serum	
6	Annexin A5	Anxa5	0050819	Negative regulation of coagulation	Anticoagulant
4	α2-u-globulin (mj urinary protein 6)	Mup6	0005550	Lipocalin-like pheromone transport	Allergen
7	Apolipoprotein E	ApoE	0017127	Cholesterol, lipid transport in serum	Sf-PhL
17	Plasma retinol binding protein	RBP4	0001972	Plasma retinol and vitamin A carrier	SARS; VA; APR (negative)
18	Retinoic acid binding protein 2	CRABP2	0001972	Retinoic acid (retinol) binding	APR; VA
22	Transthyretin	TTR	0005179	Vitamin A and T4/thyroxine transport	APR; VA

^a^ Proteins spots from 2D electrophoresis identified by mass spectrometry. ^b^ Selected Gene Ontology (GO) term. ^c^ Selected findings in lung diseases: APR, acute phase response; ARDS, acute respiratory distress syndrome; COPD, chronic obstructive pulmonary disease; IAV, influenza A virus infection; IPF, idiopathic pulmonary fibrosis; NF-κB, nuclear factor κB induced gene; SARS, severe acute respiratory syndrome caused by SARS-CoV; Sf-PhL, surfactant-phospholipid oxidation/regulation; TB, tuberculosis; VA, vitamin A (retinol/retinoic acid) transport, signaling and metabolism. Refer to text for references and discussion.

## Supplementary Materials

The following are available online at http://www.mdpi.com/1999-4915/10/12/670/s1, Table S1: Proteomics identification of proteins in murine BAL fluid from MHV-68 infection, Table S2. RT-PCR primers used in this study, Figure S1. Analysis of viral and cellular components in BAL fluid. Figure S2. One of the LC/MS-MS spectra identifying mouse Pdx6. Figure S3. Dynamics of ROS generation in MHV-68 infection of cultured cells. Figure S4. Tnfaip8l2 is a member of a conserved gene family in human and mouse.
