# Supplementary material for: Proteomics of Bronchoalveolar Lavage Fluid Reveals a Lung Oxidative Stress Response in Murine Herpesvirus-68 Infection
Source: Viruses. 2018 Nov 27;10(12):670. doi: 10.3390/v10120670 (PMC6316452; doi:10.3390/v10120670)
Supplement: Supplementary file 1 [file viruses-10-00670-s001.zip › pap4_Bortz_SuppFiG.pdf]

Fig. S1 (Supporting Information).

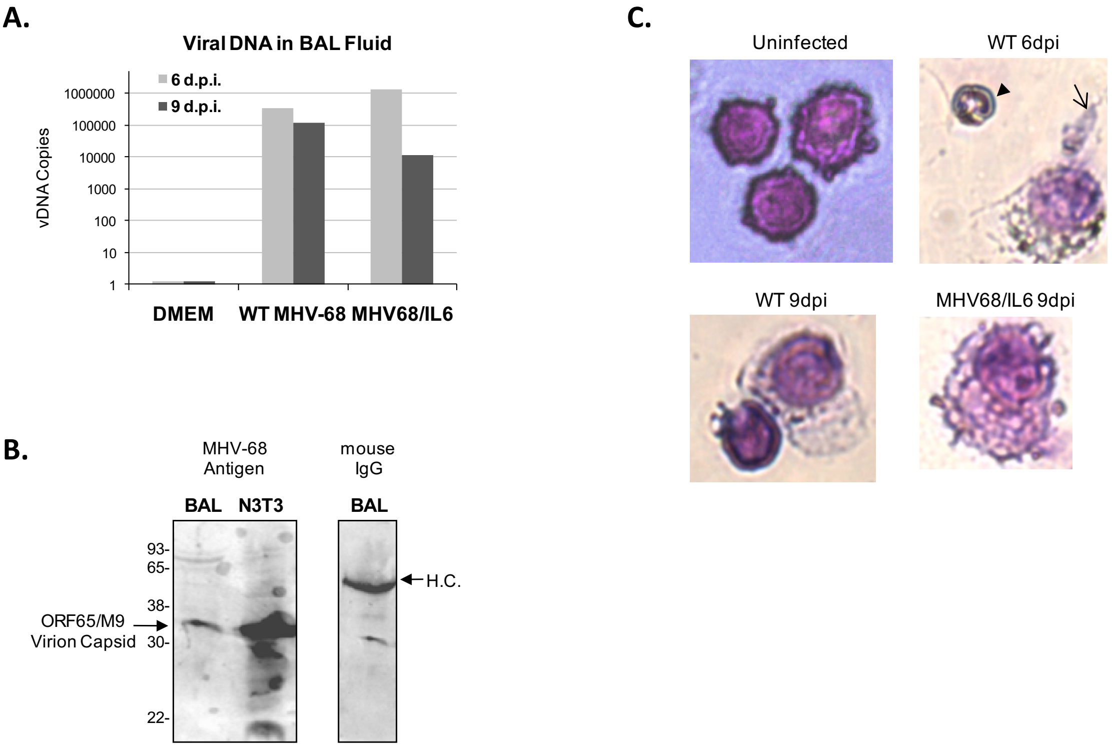

Fig. S2 (Supporting Information).

Mascot Search Results

Protein View

Match to: **Q6A0D0_MOUSE** Score: **263**
MKIAA0106 protein (Fragment).- Mus musculus (Mouse).

Nominal mass (M_r): **25168**; Calculated pI value: **5.97**
NCBI BLAST search of Q6A0D0_MOUSE against nr
Taxonomy: Mus musculus

Fixed modifications: Carbamidomethyl (C)
Variable modifications: Oxidation (M)
Cleavage by Trypsin: cuts C-term side of KR unless next residue is P
Sequence Coverage: **32%**

Matched peptides shown in **Bold Red**

```
  1 TAAMPGGLLL GDEAPNFEAN TTIGRIRFHD FLGDSWGILF SHPRDFTPVC
 51 TTELGRAAKL APEFAKRNVK LIALSIDSVE DHLAWSKDIN AYNGETPTEK
101 LPFPIIDDKG RDLAILLGML DPVEKDANNM PVTARVVFIF GPDKKLKLSI
151 LYPATTGRNF DEILRVVDSL QLTGTKPVAT PVDWKKGESV MVVPTLSEEE
201 AKQCFPKGVF TKELPSGKKY LRYTPQP
```

Start - End	Observed	Mr(expt)	Mr(calc)	Delta	Miss	Sequence
88 - 100	726.7283	1451.4421	1450.6576	0.7845	0	K.DINAYNGETPTEK.L (Ions score 31)
88 - 100	726.7283	1451.4421	1450.6576	0.7845	0	K.DINAYNGETPTEK.L (Ions score 11)
101 - 109	529.2186	1056.4227	1056.5855	-0.1628	0	K.LPFPIIDDK.G (Ions score 51)
112 - 125	771.8106	1541.6067	1541.8374	-0.2307	0	R.DLAILLGMLDPVEK.D Oxidation (M) (Ions score 92)
112 - 135	876.9840	2627.9302	2627.3298	0.6004	1	R.DLAILLGMLDPVEKDANNMPVTAR.V 2 Oxidation (M) (Ions score 43)
136 - 145	575.7542	1149.4938	1148.6593	0.8345	1	R.VVFIFGPDKK.L (Ions score 7)
148 - 165	693.6130	2077.8172	2078.1160	-0.2988	1	K.LSILYPATTGRNFDEILR.V (Ions score 38)

Peptide View

MS/MS Fragmentation of **DLAILLGMLDPVEK**
Found in **Q53ZU7_MOUSE**, **Peroxiredoxin 6.- Mus musculus (Mouse).**
Found in **Q6A0D0_MOUSE**, MKIAA0106 protein (Fragment).- Mus musculus (Mouse).
Match to Query 115: 1541.606730 from(771.810641,2+)
Title: File: sbRENSUN051506_EB5-2.wiff, Sample: Eric5-2 (sample number 1), Elution: 45.66 min, Period: 1, Cycle(s): 2235 (Experiment 2)
Data file E:\Temp\mas11.tmp

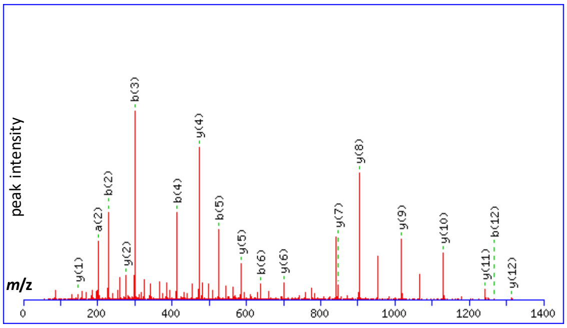

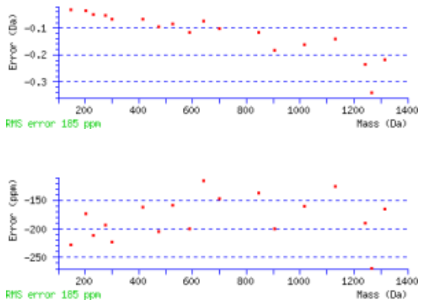

Monoisotopic mass of neutral peptide Mr(calc): 1541.8374
Fixed modifications: Carbamidomethyl (C) (apply to specified residues or termini only)
Variable modifications:
M8 : Oxidation (M)
Ions Score: 92 **Expect:** 1.9e-07
Matches : 18/104 fragment ions using 26 most intense peaks

#	a	a^{++}	b	b^{++}	Seq.	y	y^{++}	y*	y^{*++}	#
1	88.0393	44.5233	116.0342	58.5207	D					14
2	201.1234	101.0653	**229.1183**	115.0628	L	1427.8178	714.4125	1410.7912	705.8992	13
3	272.1605	136.5839	**300.1554**	150.5813	A	***1314.7337***	657.8705	1297.7072	649.3572	12
4	385.2445	193.1259	**413.2394**	207.1234	I	***1243.6966***	622.3519	1226.6701	613.8387	11
5	498.3286	249.6679	**526.3235**	263.6654	L	***1130.6125***	565.8099	1113.5860	557.2966	10
6	611.4127	306.2100	**639.4076**	320.2074	L	***1017.5285***	509.2679	1000.5019	500.7546	9
7	668.4341	334.7207	696.4290	348.7182	G	***904.4444***	452.7258	887.4179	444.2126	8
8	815.4695	408.2384	843.4644	422.2359	M	***847.4230***	424.2151	830.3964	415.7018	7
9	928.5536	464.7804	956.5485	478.7779	L	***700.3876***	350.6974	683.3610	342.1841	6
10	1043.5805	522.2939	1071.5754	536.2914	D	***587.3035***	294.1554	570.2770	285.6421	5
11	1140.6333	570.8203	1168.6282	584.8177	P	***472.2766***	236.6419	455.2500	228.1286	4
12	1239.7017	620.3545	**1267.6966**	634.3519	V	375.2238	188.1155	358.1973	179.6023	3
13	1368.7443	684.8758	1396.7392	698.8732	E	***276.1554***	138.5813	259.1288	130.0681	2
14					K	***147.1128***	74.0600	130.0863	65.5468	1

All matches to this query

Score	Mr(calc):	Delta	Sequence
92.2	1541.8374	-0.2307	DLAILLGMLDPVEK
9.3	1541.8664	-0.2597	GLELISKAIEIDNK
8.9	1540.9592	0.6475	VTVLLILVSWSLAK
8.8	1542.8268	-1.2201	IITTWIMYIFAR
8.4	1541.8202	-0.2134	LWLPTASVSPSQTR
8.2	1542.8114	-1.2047	QATLLSHTSSSQRK
8.1	1542.7638	-1.1570	LQEEDLQDLERR
7.2	1541.7362	-0.1294	AQWGSSDATPELPAL
5.6	1541.7586	-0.1519	DLGTASHNFSGALPR
4.3	1541.8241	-0.2174	QVAFHIPFEVVEK

Fig. S3 (Supporting Information).

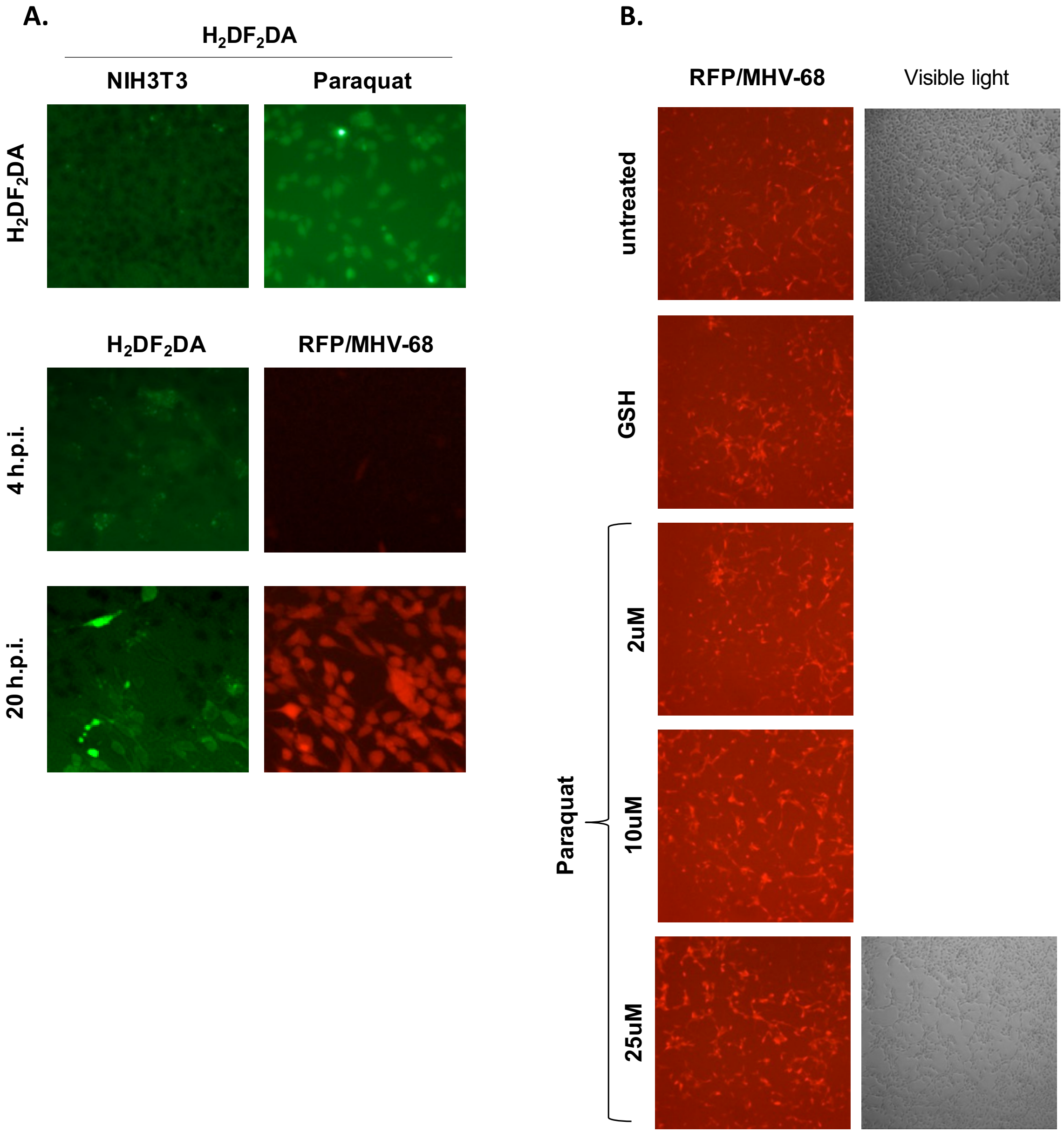

Fig. S4 (Supporting Information).

```
O95379:TFIP8_HUMAN       MHSEAEESKEVATDVFNSKNLAVQAQKKILGKMVSKSIATTLIDDTSSEVLDELYRVTRE
Q921Z5:TFIP8_MOUSE       MLSEAEEPREVATDVFNSKNLAVQAQKKILGKMVSKSIATTLIDDTSSEVLDELYRVTKE
sp:Q8WVP5:TP8L1_HUMAN    M------------DTFSTKSLALQAQKKLLSKMASKAVVAVLVDDTSSEVLDELYRATRE
sp:Q8K288:TP8L1_MOUSE    M------------DTFSTKSLALQAQKKVLSKMASKAMVAVFVDNTSSEVLDELYQATKE
sp:Q6P589:TP8L2_HUMAN    M------------ESFSSKSLALQAEKKLLSKMAGRSVAHLFIDETSSEVLDELYRVSKE
sp:Q9D8Y7:TP8L2_MOUSE    M------------ESFSSKSLALQAEKKLLSKMAGRSVAHLFIDETSSEVLDELYRVSKE
                         !            : !.:!.!!:!!:!!:!.!!..:::.  ::!:!!!!!!!!!!:.::!

O95379:TFIP8_HUMAN       YTQNKKEAEKIIKNLIKTVIKLAILYRNNQFNQDELALMEKFKKKVHQLAMTVVSFHQVD
Q921Z5:TFIP8_MOUSE       YTQNKKEAERVIKNLIKTVIKLAVLHRNNQFNQDELALMEKFKKKVHQLAMTVVSFHQVE
sp:Q8WVP5:TP8L1_HUMAN    FTRSRKEAQKMLKNLVKVALKLGLLLRGDQLGGEELALLRRFRHRARCLAMTAVSFHQVD
sp:Q8K288:TP8L1_MOUSE    FTRSRKEAQRVVKNLVKVAVKLAVLLRADQLDSNELAQLQRFRGRVRSLAMTALSFHQVD
sp:Q6P589:TP8L2_HUMAN    YTHSRPQAQRVIKDLIKVAIKVAVLHRNGSFGPSELALATRFRQKLRQGAMTALSFGEVD
sp:Q9D8Y7:TP8L2_MOUSE    YTHSRPKAQRVIKDLIKVAVKVAVLHRSGCFGPGELALATRFRQKLRQGAMTALSFGEVD
                         :!:.: :!::::!:!:!..:!:.:! ! . :.  !!!   :!: : :  !!!.:!! :!:

O95379:TFIP8_HUMAN       YTFDRNVLSRLLNECREMLHQIIQRHLTAKSHGRVNNVFDHFSDCEFLAALYNPFGNFKP
Q921Z5:TFIP8_MOUSE       YTFDRNVLSRLLNECRELLHEIIQRHLTAKSHGRVNNVFDHFSDCDFLAALYNPFGKFKP
sp:Q8WVP5:TP8L1_HUMAN    FTFDRRVLAAGLLECRDLLHQAVGPHLTAKSHGRINHVFGHLADCDFLAALYGPAEPYRS
sp:Q8K288:TP8L1_MOUSE    FTFDRRVLATGLLECRDLLHQAIGPHLTAKSHGRINHIFSHFANGDFLAALYSPAEPYRS
sp:Q6P589:TP8L2_HUMAN    FTFEAAVLAGLLTECRDVLLELVEHHLTPKSHGRIRHVFDHFSDPGLLTALYGP--DFTQ
sp:Q9D8Y7:TP8L2_MOUSE    FTFEAAVLAGLLVECRDILLELVEHHLTPKSHDRIRHVFDHYSDPDLLAALYGP--DFTQ
                         :!!:  !!:  ! !!!::! : :  !!!.!!!.!:.::!.! ::  :!:!!!.!   :

O95379:TFIP8_HUMAN       HLQKLCDGINKMLDEENI
Q921Z5:TFIP8_MOUSE       HLQKLCDGINKMLDEENI
sp:Q8WVP5:TP8L1_HUMAN    HLRRICEGLGRMLDEGSL
sp:Q8K288:TP8L1_MOUSE    HLCRICDGLGRMLDEGGI
sp:Q6P589:TP8L2_HUMAN    HLGKICDGLRKLLDEGKL
sp:Q9D8Y7:TP8L2_MOUSE    HLGKICDGLRKLLDEGKL
                         !! ::!:!: ::!!!  :
```
