# Supplementary material for: Proteomics of Bronchoalveolar Lavage Fluid Reveals a Lung Oxidative Stress Response in Murine Herpesvirus-68 Infection
Source: Viruses. 2018 Nov 27;10(12):670. doi: 10.3390/v10120670 (PMC6316452; doi:10.3390/v10120670)
Supplement: Supplementary file 1 [file viruses-10-00670-s001.zip › pap4_Bortz_SupplementaryTables.pdf]

Supporting Information Table S1. Proteomics identification of proteins in BAL fluid.

Spot(s)	BAL Protein Indetification	Symbol	Accession[1]	Number of Peptides[2]	Mass Spectrometry[3] M-Score	Z-score	Regulation[4] MHV68	MHV68/IL6
1	α1-acid glycoprotein B	A1AG1	Q60590	2	109		21.9	19.4
2	α1-anti-trypsin (serpin A1)	A1AT6	P81105	9	298		1.5	1.6
3	α2-macroglobulin	A2MP	Q6GQT1	2	25*		1.3	25.1
4	α-2u-globulin (major urinary protein 6)	Mup6	P02762	8	71-185		2.3	2.1
5	Albumin	Alb	P07724	15	n.s.	2.87, 4%	0.8	0.8
6	Annexin A5	Anxa5	Q99LA1	1-8	44-186		3.1	3.5
7	Apolipoprotein E	ApoE	P08226	14	n.d.	2.16, 7%	1.5	1.3
8	Calcyclin	S100a6	A54314	3	83		0.6	0.6
9	Clara cell protein 10	CC10	Q06318	1	50		0.8	0.7
10	Glutathione-S-transferase, mu1	GSTm1	P10649	1	54		2.0	2.1
11	Haptoglobin	Hp	Q63927	7	160		0.6	0.6
12	Hydrocephalus-inducing protein [fgt.]	Hydin	Q80W93	9	43		9.5	9.5
	IL1 family member 10	IL1f10	Q8R459	4	n.d.	2.44, 9%		
13	Oxytocin receptor [fgt.]	OxtR	P97926	2	25*		1.1	1.1
14	Peroxiredoxin 2	Pdx2	Q61171	14	98		1.3	48.9
15	Peroxiredoxin 6	Pdx6	O08709	3-32'	52-263		5.9	6.9
16	Phospholipase A2, secreted	PLA2G12A	Q9EPR2	3	n.d.	2.89, 14%	21.0	27.9
17	Plasma retinol binding protein	RBP4	Q6T898	2	96	2.17, 8%	23.6	55.9
18	Retinoic acid binding protein 2 [isoform]	CRABP2	P22935	1-2	81-83		1.3	53.1
19	Superoxide dismutase 3, extracellular	EC-SOD	P08228	1-4	76-163	3.12, 15%	3.9	82.4
20	Thioredoxin-like 4B	TXNL4B	Q8BUH1	4	n.d.	3.42, 15%	1.3	21.0
21	TNFα-induced protein 8-like 2	Tnfaip8l2	Q9D8Y7	2	23*		5.1	5.1
22	Transthyretin [major form]	TTR	Q9D6A4	3-12	84-188		8.8	10.1

Regulation Key
suppressed
no significant change
weakly induced
strongly induced

Notes:

1 UniProt Accession number (*Mus musculus*).

2 Number of matching peptides in mass spectromtery; range indicated for multiple spots/detections.

3 LC/MS-MS with highest individual Mascot peptide score: M>38, *p*<0.05 (significant);38>M>23, *p*<0.10 (*marginal significance); or MALDI with associated Z-score and % peptide sequence coverage by *Aldente*. n.d., not detected; n.s., not significant.

4 Ratio of each protein's total 2D PAGE spot density to DMEM for WT MHV-68 or MHV68/IL6 9d.p.i. in mouse BAL; normalized to average oxytocin-receptor and albumin; regulation indicated.

Supporting Information Table S2. PCR Primers Used in This Study

Mouse *or* Viral Gene	Symbol	Primer Sequences [5' to 3']	
		Forward	***Reverse***
**MHV-68 Genome**	ORF65	GTCAGGGCCCAGTCCGTA	TGGCCCTCTACCTTCTGTTGA
	ORF57	ACTGAAACCTCGCAGAGGTCC	GCACGGTGCAATGTGTCACAG
**Actin**	Actin	CACCCACACTGTGCCCATCTAC	GTGAGGATCTTCATGAGGTAGTC
**Glutathione peroxidase 3**	Gpx3	GATCCTCCGGGCATCCTGCCTTCT[illegible]	TCGAACATACTTGAGACTGGGGAGT
**Nitric oxide synth. 2, inducible**	iNOS	CTTTGTGCGGAGTGTCAGTGG	TTCTTCCTGATAGAGGTGGTCC
		ACATCGACCCGTCCACAGTAT	CAGAGGGGTAGGCTTGTCTC
**Peroxiredoxin 6**	Pdx6	CGCCAGAGTTTGCCAAGAG	TCCGTGGGTGTTTCACCATTG
		TTGATGATAAGGGCAGGGAC	CTACCATCACGCTCTCTCCC
**Retinoic acid b. protein 4**	RBP4	TGCGTTCAGGAGTGATCAAG	CCATGTCTGCACACACTTCC
**Superoxide dismutase 3**	SOD3	TTGTTCTACGGCTTGCTACTGGC	ATTGCATGCATCTCGGCAGC
		AGGTGGATGCTGCCGAGAT	TCCAGACTGAAATAGGCCTCAAG
		GGTTGAGAAGATAGGCGACAC	CGTGGCTGATGGTTGTACC
**Thioredoxin 1**	Trx1	CTGGTTCTGCTGAGACGCGT	AGAGGGAATGGAAGAAGGGCT
**TNFα-induced prot. 8-like 2**	TIPE2	TTACCGCGTGTCCAAAGAA	AGTCCACCTCACCGAAGCTA
**Transthyretin**	TTR	TGTCCTCTGATGGTCAAAGTCCT	CTGCGATGGTGTAGTGGCGAT
