# Supplementary material for: Proteomics of Bronchoalveolar Lavage Fluid Reveals a Lung Oxidative Stress Response in Murine Herpesvirus-68 Infection
Source: Viruses. 2018 Nov 27;10(12):670. doi: 10.3390/v10120670 (PMC6316452; doi:10.3390/v10120670)
Supplement: Supplementary file 1 [file viruses-10-00670-s001.zip › supplementary captions.pdf]

Supplementary Tables and Figures

Table S1. Proteomics identification of proteins in murine BAL fluid from MHV-68 infection. Proteomics identification of BAL (differentially expressed) proteins from BAL fluid from mock (DMEM), MHV-68 infected, and MHV-IL6 infected mice at 9d. p.i. Proteins spots from 2D-E were excised, digested in trypsin, and identified by MALDI and/or LC/MS-MS. Spots were also quantified by densitometry, normalized as described in Methods, to estimate fold induction over mock (DMEM) control.

Table S1 Notes:

[1] UniProt Accession number *(Mus musculus).*

[2] Number of matching peptides in mass spectromtery; range indicated for multiple spots/detections.

[3] Significance of mass spectrometry identification: LC/MS-MS, using Mascot software, listing highest individual peptide M score: M>38, $p<0.05$ (significant); 38>M>23, $p<0.10$ (*marginal significance); or MALDI, using Aldente software, listing Z-score and % peptide sequence coverage; *n.d.*, not detected; *n.s.*, not significant.

[4] Regulation of protein abundance in mouse BAL from WT MHV-68 or MHV68/IL6 at 9d.p.i. estimated by normalized ratio of each protein's total 2D-E spot density to orthologous spot in mock (DMEM) control 2D-E; regulation indicated in key.

Table S2. RT-PCR primers used in this study. Source of primer sequences from references specified or RT-PCR primer database listed.

Figure S1. Analysis of viral and cellular comnponents in BAL fluid. BAL fluid from uninfected (DMEM-inoculated), WT MHV-68-infected or MHV68/IL6-infected mice was recovered at 6 d.p.i. and 9 d.p.i., pooled, and separated by centrifugation into supernatant and cellular phases. **(a)** Viral DNA copies in BAL fluid supernatant was analyzed by quantitative PCR, averaged for 3 mice in each condition, according to reference standards; differences not significant ($p>0.1$, two-tailed t-test). **(b)** Proteins in processed BAL fluid supernatant were separated by SDS-PAGE and viral ORF65/M9 capsid protein was detected by Western blot with polyclonal antisera. NIH3T3 cells infected with WT MHV-68 provided a positive control for ORF65/M9 antisera. BAL supernatant Western blot was also probed with monoclonal HRP-labeled goat anti-mouse secondary antibody alone, detecting mouse IgG heavy chain (H.C.) as indicated. **(c)** BAL cells pelleted by low speed centrifugation were resuspended on glass slides and stained by hematoxylin-eosin. BAL cells were predominantly (>75%) mononuclear; erythrocytes *(arrowhead)* and cellular/fibrous debris or platelets *(arrow)* were also occasionally visible.

Figure S2. One of the LC/MS-MS spectra identifying mouse Pdx6. Protein spots excised from SYPRO-Ruby stained 2D-PAGE were digested in trypsin, separated by LC, and peptides analyzed on a Sciex Q-star ion trap mass spectrometer with tandem peptide ion fragmentation running in data-dependent mode. Peptide mass (*Mr* or *m*) and charge (*z*) and MS/MS fragment ions were analyzed by *Mascot* software with error tolerance <1 Da, 1 tryptic digest miss allowed, fixed

carbamamidomethyl modification of cysteine, and variable oxidation of methionine. Data were searched against the non-redundant mouse proteome identifying 7 peptides to a database isoform of mouse peroxiredoxin 6 ("Protein View") with composite protein match probability score calculated (match probability, log $p=10^{-SCORE}$). Fragment ion MS/MS spectra were displayed by *m/z* and intensity and matched to predicted a-, b- and y-series fragmentation ions ("Peptide View"). Additional fragment ion spectra matches with ion expectation scores were considered but are below significance cutoff of 38 ($p<0.05$).

Figure S3. ROS during MHV-68 infection in cultured fibroblasts. **(a)** MHV-68 does not induce ROS by 4 h.p.i. Murine NIH3T3 fibroblasts were infected (m.o.i.=5) with RFP/MHV-68 (*red channel*). Supernatants were removed at 4 or 20 h.p.i., replaced with PBS containing H_2DF_2DA, and cells imaged by epifluorescence microscopy. Paraquat (10uM) was a control for induction of ROS leading to oxidative fluorescence of H_2DF_2DA *(green channel)*. **(b)** Modulation of ROS does not drastically affect MHV-68 replication. Untreated NIH3T3 cells or cells treated with soluble glutathione (GSH), a ROS quencher, or increasing concentrations of paraquat, a ROS inducer, were infected with RFP/MHV-68 (m.o.i.=1) and imaged 20 h.p.i.

Figure S4. Tnfaip8l2 is a member of a conserved gene family in human and mouse. Amino acid sequences for human and murine homologues of TNF -induced protein 8 were retrieved by UniProt database Accessions and aligned by CLUSTAL. TP8L2_mouse is Tnfaip8l2, also called mouse TIPE2, the protein identified in this study. Similarity indicated for conserved residues (!), similar residues (:), and weakly similar residues (.).
